# Neurophysiological Activations of Predictive and Non-predictive Exogenous Cues: A Cue-Elicited EEG Study on the Generation of Inhibition of Return

**DOI:** 10.3389/fpsyg.2019.00227

**Published:** 2019-02-08

**Authors:** Ana B. Vivas, Evangelos Paraskevopoulos, Alejandro Castillo, Luis J. Fuentes

**Affiliations:** ^1^Psychology Department, The University of Sheffield International Faculty, City College, Thessaloniki, Greece; ^2^Medical Physics Laboratory, School of Medicine, Aristotle University of Thessaloniki, Thessaloniki, Greece; ^3^Department of Basic Psychology and Methodology, University of Murcia, Murcia, Spain

**Keywords:** inhibition of return, predictive cues, exogenous cuing task, EEG and ERP, intraparietal sulcus

## Abstract

In cueing tasks, predictive and non-predictive exogenous spatial cues produce distinct patterns of behavioral effects. Although both cues initially attract attention, only non-predictive cues lead to inhibitory effects (worse performance at the cued location as compared to the uncued location) if the time elapsed between the cue and the target is long enough. However, the process/processes leading to the later inhibitory effect, named inhibition of return (IOR), are still under debate. In the present study, we used cue-elicited EEG activations from predictive and non-predictive exogenous spatial cues to further investigate the neural processes involved in IOR. Unlike previous similar studies, we intermixed both types of cues in a block of trials, in an attempt to identify the unique neurophysiological activations associated with the generation of IOR. We found that predictive and non-predictive cues significantly differed in activation just at 400–470 ms post-cue window. Activation was greater for non-predictive cues in the intraparietal sulcus (IPS), and this activation correlated significantly with IOR effects. These findings support the hypothesis that the posterior parietal cortex plays a crucial role in the generation of IOR.

## Introduction

In a world crowded with information, we often need to ignore irrelevant locations and objects that get in the way of the target location/object. Experimentally, the visuospatial cueing paradigm (Posner, 1980) has been employed to study visuospatial selective processes. In this paradigm, a change in the brightness of a location (the spatial cue) has been typically used to attract attention, similarly to a neon signal attracting our attention while driving at night. To study how our attentional system deals with irrelevant information, Posner and Cohen (1984) first investigated the effect of non-predictive peripheral cues (on 50% of the trials the target would appear in the same location of the cue) on response times and accuracy in a target detection task. They found that when the interval between the spatial cue and the target was rather long (about 300 ms), responses at the previously cued –attended – location were slower as compared to a novel, non-cued, location (the inhibition of return effect – IOR; Posner et al., 1985).

More than 30 years have passed since the seminal study of Posner and Cohen and still there is not a consensus about the mechanism(s) underlying this effect. Initially, as the name of the empirical effect suggested, it was proposed that this effect results from the inhibition of attention to return to an already explored (irrelevant) location (Posner et al., 1985). The *re-orienting hypothesis of IOR* implies that since the cue is irrelevant, attention would be withdrawn after some time from that location and moved back to fixation as this would be the best strategy for speeding up target detection. The delayed response to the cued location would reflect an “inhibitory” mechanism to prevent attention from returning to the old location, in favor of new non-previously explored locations (see Klein, 2000, for a review). This account suggests that the processes leading to IOR start with the cue onset, and the following withdrawal (disengagement) of attention from the cued location.

Since this early account of the effect, other explanations have been put forward. For instance, Dukewich (2009) proposed the *habituation hypothesis of IOR*. Specifically, the author refers to early biological/physiological theories of habituation to propose that non-predictive cues, which are presented repeatedly at the same location, would lead to habituation of the orienting response. That is, they would produce a decrement in the reflexive attentional orienting response, which consequently would have detrimental effects in detecting the target. The author argues that habituation could take place at different levels, namely at the sensory (habituation of the perceptual attribute of the location) and response level (habituation of the orienting response). This hypothesis is supported by studies that have reported attenuation of the neuron responses in the Superior colliculus (SC) with repeated presentations of non-predictive cues relative to predictive ones (Fecteau and Muñoz, 2005). According to this hypothesis the process (habituation) leading to IOR begins with the onset of the cue as well. That is, the reflexive orienting response to the target at the cued location is affected because across trials the cue generates weaker reflexive orienting responses due to sensory and motor habituation.

In a similar vein, Lupiáñez (2010) has argued against a re-orienting hypothesis of IOR and proposed *the detection-cost theory* of IOR. Lupiañez and colleagues (Lupiáñez, 2010; Lupiañez et al., 2013) suggest that the exogenous (peripheral) spatial cue, in addition to eliciting an orienting response, produces other non-spatial effects. According to the authors, spatial cues would also open object-file representations, which integrate target events depending on spatiotemporal characteristics (e.g., more perceptually similar cue-target events, which are separated by a rather short time interval, are more likely to be integrated in a single object-file representation). Consequently, IOR effects would reflect a cost in rapidly detecting the target, because this new event is integrated in the object-file representation of the previous similar event (the cue). This theory implies that the mechanism/process responsible for the IOR effect does not start with the cue but with the target onset. As the authors claimed, exogenous and endogenous orienting of attention away from the cued location is neither a necessary nor a sufficient condition to observe IOR. That is, the processes leading to IOR would be independent from attentional orienting.

Other theories have proposed that IOR may result from the inhibition of oculomotor responses (Sapir et al., 1999; Taylor and Klein, 2000; Klein and Hilchey, 2011; Hilchey et al., 2014, 2016) or from a change in response criterion (Ivanoff and Klein, 2001, 2006).

All these hypotheses are not necessarily mutually exclusive, and it might be that the onset of an exogenous non-predictive cue activates several processes associated with the processing of the cue (e.g., orienting responses and selection/preparation of oculomotor and motor responses) so that the organism is more effectively biased to explore new locations. Behaviorally, we are not able to disentangle the proposed process or processes that lead to IOR, since we usually measure just the response delay associated with detecting targets presented at the previously cued location. One way to get around this problem is to use cue-elicited EEG-ERP measures, so that we are able to study the physiological/neural processes that lead to the IOR effect. So far, the majority of the ERP studies conducted on IOR have investigated target-locked processes. Although there are inconsistencies across studies, most of them have focused on early ERP components, e.g., P1 and N1. That is, several studies have reported a reduced P1 component in the cued compared to the uncued location (Hopfinger and Mangun, 1998; Doallo et al., 2004; Prime and Ward, 2004, 2006; Wascher and Tipper, 2004). The P1 modulation associated with IOR would reflect reduced sensory processing of the target at the inhibited location. However, the finding of reduced P1 has not been linked with behavioral IOR in all the studies (Eimer, 1994; Hopfinger and Mangun, 1998; Hopfinger and Mangun, 2001; Doallo et al., 2004). In addition, there is also a reduction of the N1 component in the cued location condition when more complex discrimination tasks are employed (Amenedo et al., 2014; Gutiérrez-Domínguez et al., 2014). Finally, target-locked EEG studies have also reported a delay of response selection/preparation processes at the cued location. For instance, Pastötter et al. (2008) found reduced ERD (Event-Related Desynchronization) in the beta band for targets presented at the cued location, and this reduction was cue-dependent. The target-locked findings agree with the hypothesis that IOR may reflect inhibition of different processes, sensory/attentional and motor.

As far as we know, only three studies have investigated EEG activity locked to the cue in an IOR procedure (Amenedo et al., 2014; Tian et al., 2011; Wascher et al., 2011). Both Amenedo et al.’s (2014) and Wascher et al.’s (2011) studies compared two age groups (younger and older adults) to investigate age-related changes in processing non-predictive cues. Wascher et al. (2011) found that older adults had smaller frontocentral (FCz) N2 amplitudes than younger adults, and this finding was significantly associated with later onset of IOR. On the other hand, Amenedo et al. (2014) reported that older adults exhibited overall more negative cue-locked mean amplitudes, and this negativity was maximal at centro-parietal and parieto-occipital electrodes. As suggested by Amenedo et al. (2014), these two apparently contradictory findings can be combined into a single explanation. That is, older adults would engage more attentional resources to process the cue (greater negative mean amplitudes), and consequently fail to effectively ignore the cue at shorter SOAs (lack of N2 inhibitory component in the Wascher et al. (2011) study). More relevant to the present work is the study of Tian et al. (2011), which investigated cue-locked EEG activity in young adults as a function of cue-target SOA. They argued that since participants do not know in advance if the cue is valid or invalid, the cue-locked activation for these two conditions should not differ. Thus, the authors compared activations in short versus long SOA trials, assuming that activation found at short trials reflects facilitatory processes whereas activation found at long SOA trials reflects inhibitory processes (IOR). In the early stage of cue processing (110–240 ms), the authors found significant activation (C1, P1, and Nc components) located in primary visual cortex areas. Late P1 and Nc components located in the occipito-parietal and frontal areas were also found at this stage. In the middle processing stage (240–350 ms), they key stage for the generation of IOR, they found greater activation (P3 and Nc components) in the posterior parietal cortex, and weaker activation in the prefrontal cortex. Based on these findings, the authors proposed a neurocognitive model of IOR where the prefrontal cortex would send a signal to the SC in early stages of cue processing (top-down modulation of sensory processing of the cue). This signal would then be transferred to the posterior parietal cortex, which would generate a tag at the location of the non-predictive cue yielding the observed behavioral IOR effects.

In the present study, we focused on cue-elicited EEG activation to investigate the processes leading to IOR, but we used a rather different approach to that of previous studies. Tian et al. (2011) study was informative with regard to brain networks activated at different processing stages of the irrelevant exogenous cue. However, because irrelevant exogenous cues activate both facilitatory and inhibitory processes, it cannot be conclusively determined which activation was unique to the emergence of the inhibitory (IOR) component. The authors assumed, based on the behavioral time course of IOR, that activations at the 240–350 ms post-cue interval would be responsible for the emergence of IOR. However, several studies suggest that inhibition and facilitation might be activated in parallel, and thus may overlap in time (e.g., Posner and Cohen, 1984; Kalogeropoulou et al., 2015). To disentangle the activation associated with facilitatory and inhibitory processes elicited by the cue, and identify the unique activation associated with IOR, in the present study we compared cue-elicited activation for exogenous predictive vs. non-predictive cues within the same block of trials. The present design and analyses may also help to indirectly test some of the aforementioned theories on IOR, and contribute to a better understanding of what are the processes leading to IOR.

We make the following general predictions based on the theories discussed above. With regard to behavioral data, we expect to find facilitation with predictive cues and IOR with non-predictive cues. According to the habituation hypothesis (Dukewich, 2009) and to studies investigating the response of neurons in the SC to non-predictive cues (see Fecteau and Muñoz, 2005); we expect that non-predictive cues will elicit significantly smaller activation, as compared to predictive cues, in areas subserving attentional orienting responses (the posterior parietal cortex). On the contrary, according to theories that propose inhibition of attentional re-orienting to the cued location (Posner’s re-orienting hypothesis) or reduced saliency of the cued location in a spatial map contained in the parietal cortex (Vivas et al., 2003, 2006; Fuentes, 2004); we expect greater activation in the posterior parietal cortex in the non-predictive cue condition relative to the predictive cue condition. Note that these two hypotheses would predict opposing results in relation to activation of the parietal lobe. This may lead to a rather non-falsifiable prediction, as irrespective of what result is observed one or the other account will be supported. However, falsifiability is not an issue here because most studies on brain mechanisms underlying IOR involve the posterior parietal lobe. Thus, this brain area is expected to be of special relevance for any account of IOR and constitutes the region of interest for the present study. It is not clear what predictions might follow from the detection-cost hypothesis. Since this hypothesis proposes that the response delay usually observed in IOR tasks reflects processes activated by the target onset, which are unrelated to the orienting response to the cue. We therefore hypothesize that this theory would predict no effect (null hypothesis).

In Experiment 1, we aimed to elicit IOR in a context where the predictiveness of the peripheral cues is manipulated trial-by-trial within a block and flagged by the color of the cue (red = 80% validity; blue = 50% validity). In line with previous studies that have done within block manipulations of parameters such as SOA to investigate the time course of facilitation and inhibition effects in cueing tasks, so that participants would not be able to anticipate the onset of the target and adopt different strategies, we preferred to manipulate the predictiveness of the cue within a block. In this way, we intended to make predictive and non-predictive cue conditions more comparable to ensure that participants would not adopt different task-related strategies for these conditions. We expected that predictive and non-predictive cues presented intermixed in a block of trials would produce the standard behavioral effects; facilitation with predictive cues and inhibition (IOR) with non-predictive ones. In Experiment 2, we ran the task with a different group of participants to investigate cue-elicited EEG activity.

## Materials and Methods

### Participants

Twenty-one undergraduate students (age range from 19 to 24 years old; 5 males), and a different group of 20 undergraduate students (age range from 19–25 years old; 6 males) from the University of Murcia volunteered to participate in Experiment 1 and 2, respectively. They all had normal or corrected-to-normal vision.

Both experiments were approved by the University of Murcia’s Ethics Committee and conformed with the Declaration of Helsinki for human research. Informed consent was obtained from all the participants.

### Materials and Stimuli

The experiments were designed and presented electronically using E-Prime 2.0 software (Psychology Software Tools, Pittsburgh, PA, United States). The viewing distance was approximately 70 cm from the monitor, and all stimuli appeared on a solid black background. Instruction texts were displayed in white 12- and 18-point Courier New Font. Alphanumeric stimuli (i.e., +, -) were displayed in white 45-point Courier New font. Participants were instructed to maintain their eyes at fixation throughout the experiment. The boxes, subtending visual angles of 3.6° in height by 3.6° in width, were presented 15.0° from the top of the screen and were separated by 6.4° of visual angle from center to center. The target was a white asterisk presented inside one of the boxes. Participants were asked to indicate the location of the target (left or right) by pressing the “z” and “m” keys of the keyboard. Experiment 1 and 2 differed only on the SOA values included. Also, EEG activity was recorded just in Experiment 2.

Electroencephalography activation was recorded using 32 scalp channels mounted onto an elastic cap (actiCAP, Brain Products GmbH), according to the 10–20 international system. Two additional electrodes were attached over the left and right mastoids for reference. The EEG signal was amplified (Brain Amp, Brain Products GmbH), digitized (1000 Hz sampling frequency), and filtered (1–30 Hz band-pass with a 50 Hz notch filter). The electrode impedance was kept below 5 kΩ. Vertical eye movements were monitored with supra and infra orbital electrodes.

### Procedure

**FIGURE 1:**
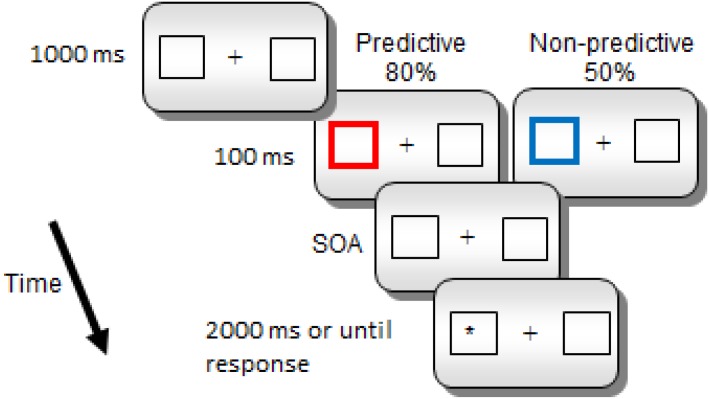
Sequence of events for a sample trial in Experiments 1 and 2.

Each trial began with a fixation point (a cross) presented in the middle of the screen for 1000 ms (see Figure 1). The fixation point was followed by two white boxes, and a central cross for 1000 ms. Then, one of the peripheral boxes became thicker and changed color, red (RGB = 255,0,0) for predictive cues and blue (RGB = 0,0,255) for non-predictive cues, for 100 ms (the spatial cue). Notice that the color-cue association was not counterbalanced, since we did not expect any potential difference in saliency related to the specific physical parameters of the colors to have any effect on the later orienting components that we examined in the study. If any, the color association effect should be expected in early rather than in late ERP components. After a further interval (SOA of 800, 1400, or 2000 ms in Experiment 1 and 800 or 1400 ms in Experiment 2) the target (a white asterisk) appeared inside one of the two lateral boxes. The target remained visible either until a response was made or 2000 ms had elapsed without any response. There was one practice block of 10 trials, and two experimental blocks of 300 trials each. Overall, there were 300 trials for the predictive condition; in 240 of these trials the target appeared in the cued location whereas in the remaining 60 trials, it appeared in the uncued location. Also, there were overall 300 trials for the non-predictive condition; in 150 of these trials the target appeared in the cued location, while it appeared in the uncued location in the other half of trials. For each cue condition, there were equal numbers of trials for each SOA condition.

### Statistical Analyses

Statistical analyses for the behavioral data were performed using SPSS v19.0 (SPSS, Inc., Chicago, IL, United States). All alpha levels were set at *p* < 0.05. Reaction times (RTs) from correct responses were submitted to a repeated measures ANOVA with cue predictiveness (predictive vs. non-predictive), SOA (800, 1400, 2000 ms for Experiment 1, and 800, 1400 ms for Experiment 2), and target location (cued and uncued) as within-subject factors. There were no anticipatory responses, and 1% of the reaction times trials were eliminated from the analyses due to incorrect responses (overall accuracy for both experiments was 99%). Bonferroni comparisons were conducted for significant main effects of SOA. For analyzing significant interactions, we conducted hypothesis driven paired samples *t*-tests.

### EEG Data Analysis Methods

The Brain Electrical Source Analysis software (BESA research, version 5.3.7, Megis Software, Heidelberg, Germany) was used for the processing of the EEG data. Firstly, the EEG data were re-referenced offline using a common average reference. Artifacts due to blinks or eye movements were corrected by applying an adaptive artifact-correction (Ille et al., 2002). The recorded data were separated in epochs of 1000 ms including a pre-stimulus interval of 200 ms. Epochs were baseline corrected using the interval from -100 to 0 ms. Data were filtered offline with a high pass filter of 1 Hz, a low pass of 30 Hz and an additional notch filter at 50 Hz. Epochs containing signals larger than 120 μV in the EEG were considered artifact contaminated and excluded from averaging. Separate averages were computed synchronized to the predictive and non-predictive cue conditions. All the participants and conditions were below the threshold of 20% for excluded trials. Consequently, there were no participants or conditions eliminated from the analyses.

In order to present the differences between predictive and non-predictive conditions unrestricted from the assumptions of any specific source analysis model, we conducted a statistical analysis of the fluctuation of the electric field in sensor space using BESA statistics, which included all the electrodes in the complete ERP time window. A spatiotemporal non-parametric permutation test for paired samples was applied (1000 permutations) using a cluster Alpha level of *p* < 0.05; the distance indicating neighbor sensors was set at 4 cm.

Current density reconstructions (CDR) were calculated on the neural responses of each participant for each stimulus category using the sLORETA method (Pascual-Marqui, 2002) as provided by BESA. This method is an un-weighted minimum norm that is standardized by the resolution matrix. Hence, it has the advantage of not needing an *a priori* definition of the number of activated sources. A time window of 70 ms was used for the CDR (400–470 ms). The appropriate time window was determined on the basis of the significant results of the spatiotemporal sensor space analysis as proposed by Gross et al. (2013). Each individual’s mean CDR image over the selected time-window was calculated and projected onto a standard MRI template based on the Montreal Neurological Institute (MNI) template. The images were smoothed, and their intensities normalized by convolving an isotropic Gaussian kernel with 7 mm full width half-maximum (FWHM) through Besa’s smoothing utility.

Statistical Parametric Mapping 8 (SPM8 ^1^) was used for the statistical analysis of the CDRs. Specifically, using the second level of analysis of SPM, a paired *t*-test was used to compare the responses to the cue-synchronized conditions (Predictive and Non-predictive) using the magnitude of the behavioral IOR effect (RTs_Non_predictive_Cued_-RTs_Non_predictive_Uncued_) as a covariate. Results were then constrained in gray matter using a mask, thereby keeping the search volume small and in physiologically reasonable areas. A permutation method for peak – cluster level error correction (AlphaSim) at *p* < 0.05 was applied for this whole head analysis, as implemented in REST software (Song et al., 2011), by taking into account the significance of the peak voxel (threshold *p* < 0.005 uncorrected) along with the cluster size (threshold size > 523 voxels), thereby controlling for multiple comparisons. The smoothness value used for the AlphaSim calculation was based on the smoothness of the residual image of the statistical analysis as proposed by Nichols (2012).

## Results

### Experiment 1

We found significant main effects of SOA, *F*(2,40) = 75.22, *p* < 0.0001, ηp^2^ = 0.790 and the following significant interactions: cue predictiveness × SOA; cue predictiveness × target location; and cue predictiveness × SOA × target location, *F*(2,40) = 3.28, *p* = 0.048, ηp^2^ = 0.141; *F*(1,20) = 15.86, *p* < 0.001, ηp^2^ = 0.442 and *F*(2,40) = 4.04, *p* = 0.025, ηp^2^ = 0.168, respectively (see Table 1). The analysis of the interaction cue predictiveness by target location showed significant facilitation with predictive cue [an effect of -16 ms; *t*(20) = 3.13, *p* = 0.005] and significant IOR with non-predictive cues [an effect of 7 ms; *t*(20) = 2.49, *p* = 0.022]. Furthermore, the analysis of the three-way interaction showed that with predictive cues there were significant facilitatory effects for the 1400 and 2000 ms SOA conditions [*t*(20) = 4.14, *p* = 0.001 and *t*(20) = 2.95, *p* = 0.008, respectively]; whereas for the non-predictive cues although there was a tendency for inhibitory effects at all SOA conditions, IOR effect was statistically significant only for the 1400 SOA condition [*t*(20) = 2.27, *p* = 0.034].

**Table 1:** Mean response times in milliseconds (and Standard Deviations), and mean accuracy in percentages as a Function of Cue, SOA, and Target Location in Experiment 1.

Cue	SOA	Cued	Uncued	Cued – Uncued
Predictive	800	413 (60)	421(58)	-8
		99 (0.75)	99 (1.79)	
	1400	382 (65)	406 (60)	-24^∗^
		99 (1.07)	99 (3.50)	
	2000	374 (56)	390 (54)	-16^∗^
		99 (0.70)	98 (3.34)	
Non-predictive	800	427 (59)	421 (58)	6
		99 (0.60)	99 (1.49)	
	1400	399 (61)	388 (53)	11^∗^
		99 (0.96)	99 (1.12)	
	2000	382 (52)	378 (56)	4
		99 (0.92)	99 (0.92)	

*^∗^p < 0.05*.

The analyses of mean accuracy yielded a significant main effect of location, *F*(1,20) = 5.106, *p* = 0.035, ηp^2^ = 0.203. Mean accuracy was higher in the cued location (0.995) than in the uncued location (0.990). No other effects or their interactions yielded statistically significance.

### Experiment 2

#### Behavioral Results

Results showed significant main effects of SOA, *F*(1,18) = 76.898, *p* < 0.0001, ηp^2^ = 0.810 and location *F*(1,18) = 4.405, *p* = 0.050, ηp^2^ = 0.197 (see Table 2). In addition, the cue predictiveness by location interaction reached statistical significance, *F*(1,18) = 7.864, *p* = 0.012, ηp^2^ = 0.304. The interaction was due to a significant facilitatory effect with predictive cues (Cued_mean_ = 330 ms and Uncued_mean_ = 345 ms), *t*(18) = 3.296, *p* = 0.004; whereas the inhibitory effect did not reach statistical significance for non-predictive cues (Cued_mean_ = 339 ms and Uncued_mean_ = 337 ms), *p* > 0.05. The three-way cue predictiveness × SOA × location interaction did not reach statistical significance, *F*(1,18) = 1.870, *p* = 0.188, ηp^2^ = 0.094.

**Table 2:** Mean Response Times in milliseconds (and Standard Deviations) and mean accuracy in percentages as a Function of Cue, SOA, and Target Location in Experiment 2.

Cue	SOA	Cued	Uncued	Cued – Uncued
Predictive	800	344 (43)	366 (43)	-22^∗^
		99 (0.52)	0.99 (2.81)	
	1400	316 (39)	323 (29)	-7
		99 (0.76)	99 (2.67)	
Non-predictive	800	354 (46)	354 (42)	0
		99 (0.83)	99 (2.34)	
	1400	325 (41)	321 (32)	4
		99 (1.12)	99 (2.02)	

*^∗^p < 0.05*.

The analyses of mean accuracy did not yield any significant effects, *p*s > 0.05.

In order to test whether learning of the cue color-validity contingency may have affected the time course of facilitation and inhibition effects, we conducted a 2 × 2 × 2 × 2 repeated measures ANOVA with block (1 and 2), cue predictiveness, SOA and location as the within subject factors. The main effect of block was significant, *F*(1,18) = 7.597, *p* = 0.013 (343 and 333 ms for B1 and B2, respectively), but this factor did not interact with any of the other factors.

#### EEG Sensor Space Results

**FIGURE 2:**
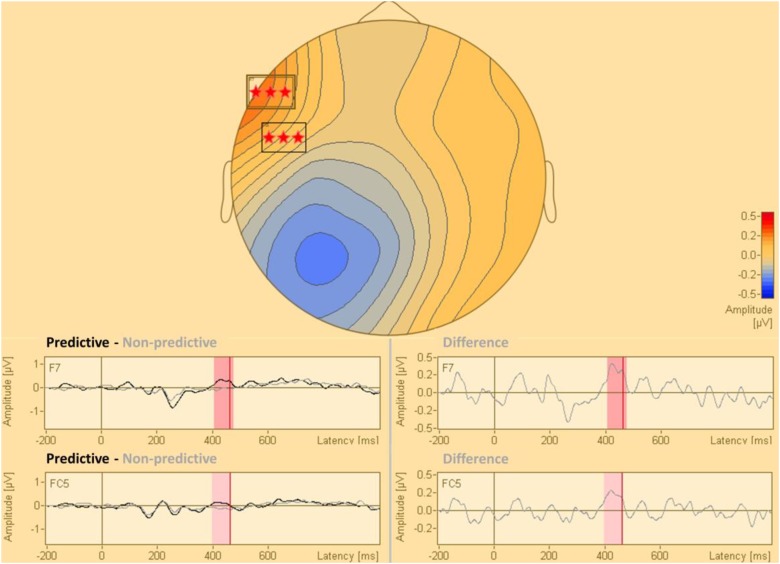
Statistical difference map (predictive vs. non-predictive) of the distribution of electric current in sensor space. The stars indicate clusters of sensors where the difference between the predictive and the non-predictive condition is significant according to the permutation tests at a significance level of *p* = 0.0001.

The statistical analysis of the cue-synchronized EEG data in sensor space, with all the sensors and in the complete time window, revealed that the two conditions (predictive and non-predictive) differed significantly in two sensors both located in the time-interval of 400–470 ms after the cue onset. This difference was located in sensors f7 and fc5. The results of this analysis are presented in Figure 2.

#### EEG Source Space Results

**FIGURE 3:**
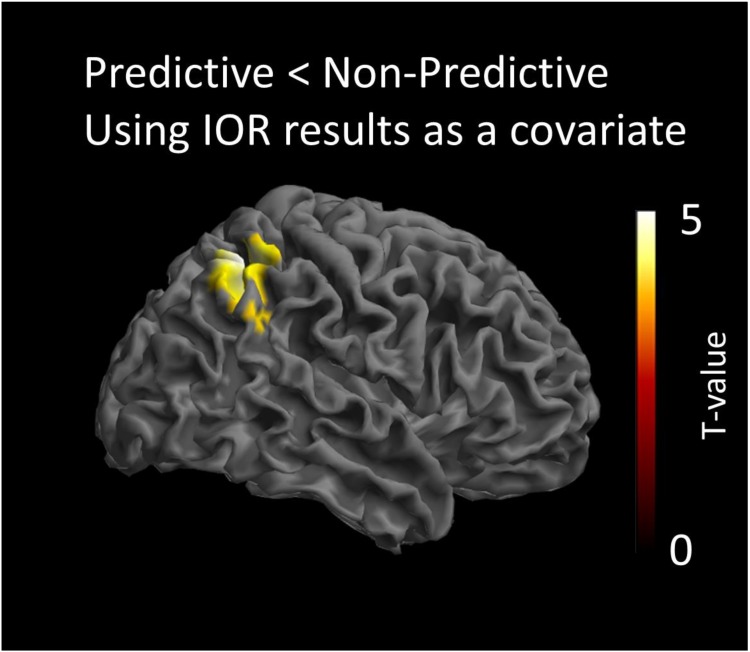
Statistical parametric maps of the cortical responses for the contrast predictive vs. non-predictive condition, as revealed by the paired *t*-test analysis using behavioral IOR effects as a covariate. Threshold: AlphaSim corrected at *p* < 0.05 by taking into account peak voxel significance (threshold *p* < 0.005 uncorrected) along with cluster size (threshold size > 523 voxels).

The statistical analysis modeling the processing of the non-predictive cue, was conducted by testing the interaction of the IOR effect with the contrast predictive < non-predictive cues. This analysis indicated that the non-predictive cue activated a region in the right intraparietal sulcus (IPS) [peak coordinates: *x* = 40, *y* = -52, *z* = 54; *t*(16) = 4.42; cluster size = 1329 voxels; *p* < 0.05 AlphaSim corrected]. This indicates that there is a significant and positive correlation of the behavioral IOR effect magnitude (RTs_Non_predictive_Cued_-RTs_Non_predictive_Uncued_) with the activity of this cortical region in the contrast of Predictive < Non-predictive. The opposite contrast modeling the interaction of the covariate with the condition Predictive > Non-predictive cue, did not yield statistically significant results. All anatomical regions were defined using the AAL atlas (Tzourio-Mazoyer et al., 2002). The statistical map of this analysis is presented in Figure 3.

## Discussion

In the present study we investigated the neurophysiological processes associated with attentional orienting to predictive and non-predictive cues using EEG. The novel aspect of the current study is that we employed a paradigm to compare behavioral and neurophysiological effects of predictive vs. non-predictive cues within the same block of trials. Thus, by comparing cue-elicited EEG responses to the two types of cues we were able to study the unique neurophysiological processes associated with non-predictive exogenous cues, which might be responsible for the behavioral effect of IOR. In line with our predictions, in Experiment 1 we found that predictive cues elicited long-lasting significant facilitatory effects (response times were faster at the cued location relative to the uncued location) at the 1400 and 2000 ms SOAs; whereas non-predictive cues yielded a significant inhibitory effect (IOR) only at the 1400 SOA. In Experiment 2, we replicated the significant cue by location interaction. That is, there was a significant facilitatory effect with predictive cues, which was eliminated with non-predictive cues. However, in this experiment we failed to observe a significant IOR effect.

The present study shows that IOR effects can be elicited in a procedure in which highly predictive and non-predictive cues are intermixed, and the validity of the cue is flagged by its color (Experiment 1). However, the inhibitory effect was not as robust; since it was no longer significant at the 2000 ms SOA in Experiment 1 or in Experiment 2. Also, the onset of IOR was rather late (at the 1400 ms SOA in Experiment 1) as compared to typical cueing studies. The findings of a less robust effect with later onset could be explained by two specific characteristics of the procedure: (i) the intermixing of predictive and non-predictive cues, which might have affected the overall perceived relationship between the cue and the target (in most trials the target was presented in the cued location), and (ii) the rather long SOA ranges employed. Previous research has shown that the time course and magnitude of faciliatory and inhibitory effects in cueing tasks is affected by parameters such as the SOA range (Cheal and Chastain, 2002), the difficulty of the task (Lupiáñez et al., 1997) and the cue-target location predictability (Wright and Richard, 2000), since changes on those parameters may influence the strategic endogenous allocation of attention to the cue, and the overall attentional control setting (Klein, 2000). For instance, Wright and Richard (2000) did not find IOR effects in a low-validity condition (the target appeared at the cued location on 10% of the trials and at the opposite uncued location on 10% of the trials), where the cue was informative though of the most probable location of the target (the target appeared at the distractor central location on 80% of the trials). The authors concluded that in situations where the cue is informative of the most probable location of a target (low or high validity conditions), inhibition is not activated or is masked by top-down prolonged facilitation. In other words, they claimed that IOR is modulated by the participant’s knowledge and strategic focus of attention regardless of cue validity. In our study, as pointed above, the target appeared in the cued location on the majority of the trials (on 65% of the trials of a block), so perhaps participants focused their attention strategically at the cued location, leading to prolonged facilitation in both cue conditions. Lupiáñez et al. (1997) also found that IOR appeared later and decayed faster on more difficult discrimination tasks. That is, when the task is more difficult (e.g., processing demands) participants adopt a higher attentional control setting (see also Klein, 2000), which also lead to greater focused attention at the cued location and prolonged facilitation. Thus, it could also be that having the cue condition intermixed in a block created a more demanding task where participants had to switch their expectations back and forward based on the cue’s color. This might have led to a higher overall attentional setting in this task, and consequently greater in magnitude and prolonged facilitatory effects. These two hypotheses may explain why the IOR appeared late and was rather small in magnitude. Finally, the differences in SOA range between Experiments 1 and 2 (the SOA range was longer in Experiment 1 than in Experiment 2), may also explain why IOR was no longer significant in Experiment 2. Specifically, Cheal and Chastain (2002) found that SOA range was critical for the onset of IOR, as the effect appeared earlier with longer SOA ranges. Thus, it could be that all the three factors mentioned above (complex task, target appeared at the cued location on the majority of trials, and shorter SOA range) could have interacted in an unexpected way, in Experiment 2, to produce longer lasting and greater facilitatory effects that masked any inhibitory effects activated at the cued location.

The ERPs analyses showed a significant difference in activation between the predictive and non-predictive condition only at about 400 ms post-cue onset (400–470 ms). The sLORETA localizations, illustrated in Figure 3, showed that, at that temporal window, activation was significantly greater for non-predictive than for predictive cues in the right IPS. Importantly, results also showed a significant and positive correlation of the behavioral IOR effect magnitude (RTs_Non_predictive_Cued_-RTs_Non_predictive_Uncued_) with the activity of that cortical region in the Predictive < Non-predictive contrast. Thus, although we did not find a significant IOR effect with the behavioral data in Experiment 2, the aforementioned positive correlation strongly suggests that activation in the IPS was driven by the magnitude of IOR. The Predictive > Non-predictive contrast did not yield significant results. In other words, predictive cues did not produce differential brain activation.

The finding of greater activation in the right IPS for non-predictive relative to predictive cues fits well with the ERPs findings reported by Tian et al. (2011) and with findings from brain-damaged patients (Bartolomeo et al., 1999; Vivas et al., 2003, 2006), neuroimaging (Rosen et al., 1999; Lepsien and Pollmann, 2002), and brain stimulation (TMS) studies (Bourgeois et al., 2013). For instance, Vivas et al. (2003, 2006) found significant IOR for contralesional targets and lack of significant IOR for ipsilesional targets in patients with damage to the posterior parietal cortex. The authors concluded that the posterior parietal cortex would be responsible for tagging cued (irrelevant) locations by reducing their relative saliency in a spatial map represented in this brain area. Similarly, early studies using fMRI (Rosen et al., 1999; Lepsien and Pollmann, 2002; Chen et al., 2010) reported activation in the frontal eye fields, superior parietal cortex and anterior motor areas and not in the superior colliculus (SC). More recent studies with brain stimulation techniques (rTMS) have studied more precisely the role of specific areas within the parietal cortex in the generation of IOR. Thus, Bourgeois et al. (2013) observed that rTMS over the right IPS or right temporo-parietal junction (TPJ) abolished manual IOR for right sided targets; whereas manual and saccadic IOR for left sided targets was eliminated after rTMS only over the right IPS. Thus, particularly the IPS seems to be a key cortical area in the generation of IOR. This conclusion fits well with the correlation between the IPS activation and the magnitude of the IOR effect found in the present study.

More relevant to the methodology used in this study, Tian et al. (2011) analyzed cue-elicited ERPs with non-predictive cues (50% validity) in different post-cue temporal windows based on the assumption that neural processes in short intervals would be responsible for the observed early facilitatory behavioral effects; while neural processes in the middle temporal window (240–350 ms) would be responsible for the generation of IOR. In the early time window, the authors found activation (C1, P1, and Nc) in the primary visual cortex. Later (240–350 ms), in the window were presumably IOR is generated, they found activation in the posterior parietal cortex (PPC) and weaker activation in the prefrontal cortex. Similarly to Vivas et al. (2003, 2006), they concluded that the activation in the PPC would be responsible for the generation of IOR, that is for *tagging* the cued – irrelevant – location in a spatial attentional map. In their model of IOR, the authors proposed that an inhibitory tag is generated at the midbrain (SC), which is then transmitted to the parietal cortex. Further, they proposed a four-system model involving a control system (PEF/FEF), a planning system (PPC/PFC), a command system (SC), and the visual system. That is, connections between the prefrontal cortex, the midbrain and the posterior parietal cortex would be responsible for the spatial and timing properties of the inhibitory tagging of cued irrelevant locations.

In our study, unlike Tian et al. (2011), we contrasted activation elicited by predictive vs. non-predictive cues and found that there were no significant differences until about 400 ms post-cue. This finding confirms the hypothesis that both cues trigger a similar initial attentional orienting response. This is also supported by the lack of significant differential activation of the predictive cue in our study. Non-predictive cues produced differentially greater activation, relative to predictive cues, only in the IPS at about 400 ms post-cue. The findings of greater activation for non-predictive cues in the IPS, and lack of significant differences between the two types of cues early on (initial orienting response) do not seem to support the habituation hypothesis proposed by Dukewich (2009). As we anticipated in the Introduction, this hypothesis would predict that habituation of the orienting attention response to non-predictive cues would lead to an overall significantly smaller activation in areas subserving attentional orienting responses (posterior parietal cortex) as compared to predictive cues. On the other hand, the results are in accordance with the hypotheses that propose inhibition of attentional re-orienting to the cued location or reduced saliency of the cued location in a spatial map contained in the parietal cortex. In addition, our results do not seem to support the detection-cost hypothesis, which suggests that behavioral IOR reflects processes that take place only with the presentation of the target, since we found a differential cue-locked activation that could be linked to IOR. Future studies need to be carried out to test directly these hypotheses by for instance measuring cue-elicited EEG activations for repeated vs. non-repeated exogenous non-predictive cues.

While the current report presents a novel approach to cue-elicited EEG analyses which is data driven and free of assumptions, and so less likely to yield type I errors (Maris and Oostenveld, 2007; Maris, 2012), it may make our findings less comparable to previous studies with EEG in IOR, which have mostly focused on particular electrodes based on ROIs. Finally, one limitation of the study is that it was designed to investigate cue-elicited ERPs, and so we did not include enough trials to allow for a good SNR in order to conduct target-elicited ERPs analyses with the full factorial design (e.g., non-predictive cued vs. non-predictive uncued).

## Conclusion

We investigated the neural processes linked to non-predictive cues. Many studies have sought for a neural marker of the increased response times at the cued location (relative to the uncued location) with non-predictive cues, but findings with target-locked ERPs are not conclusive. Only few studies have looked at the cue-elicited EEG activation instead, either by comparing younger vs. older adults (e.g., Amenedo et al., 2014) or short vs. long post-cue time intervals (e.g., Tian et al., 2011). To our knowledge, this is the first study that investigated cue-elicited EEG activation by comparing predictive vs. non-predictive cues in the same block of trials, allowing us to identify what are the unique neural processes associated with non-predictive cues. We found that predictive and non-predictive cues significantly differed in activation only at the 400–470 ms post-cue window. Localization analyses also showed that activation was greater for non-predictive cues in the IPS, and that this activation was specifically driven by IOR effects. In line with previous brain-damaged (Vivas et al., 2003, 2006) and brain-stimulation (Bourgeois et al., 2013) studies, our findings suggest that the IPS plays a crucial role in the generation of behavioral inhibitory effects produced by non-predictive cues. We did not find differential activation for non-predictive cues in the prefrontal cortex, and we believe that this is in line with previous findings that suggest that although the prefrontal cortex (FEF) may play a role in producing the motor effects associated with IOR, the attentional inhibitory tag is most likely generated in the posterior parietal cortex (Dorris et al., 2002). A limitation of the present study is that we did not find a significant behavioral IOR in Experiment 2. However, behavioral IOR effects were significantly related with the cue-locked activity of the contrast predictive < non-predictive. Another limitation is the use of a 32 channel EEG system for the source reconstruction of the activity. Low-density arrays are more prone to localization errors than higher-density recordings and may impact the outcome of the source analysis (Michel et al., 2004; Sohrabpour et al., 2015). Nonetheless, it has to be mentioned that low-density EEG recordings are often used in cognitive paradigms in which the contribution of deep sources is not assumed (Moont et al., 2012; Lawrence et al., 2014), as the reconstruction accuracy and precision of the sLORETA method are consistently high in the case of a single active source even with a small number of electrodes (Saha et al., 2015). In addition, a localization error due to the small amount of EEG channels would be randomly placed and would affect each individual participant’s data set differently, not surviving the statistical analysis of the group data. Hence, as the outcome of the statistical analysis of the group data of the present study is limited to one source, we assume that this region reconstructs the source of the cortical activity accurately. Nonetheless due to the low-density EEG recording, contribution of additional sources, along with the observed one, cannot be excluded. To conclude, our study suggests that *increased activation in the IPS* is the neurophysiological trademark of non-predictive cues. A finding that fits well with the hypothesis of IOR being represented, at the neural level, in a spatial map contained in the posterior parietal cortex (Vivas et al., 2003, 2006; van Koningsbruggen et al., 2010; Tian et al., 2011).

## Author Contributions

ABV and LJF conceived and designed the study. AC programmed and controlled the EEG experiments, and ran the two experiments. ABV and AC carried out the statistical analyses of behavioral data. EP conducted the analysis of EEG data. ABV, EP, and LJF interpreted the statistical analysis results. ABV drafted the manuscript. All authors approved the final version of the manuscript for submission.

## Conflict of Interest Statement

The authors declare that the research was conducted in the absence of any commercial or financial relationships that could be construed as a potential conflict of interest.

## Footnotes

1 http://www.fil.ion.ucl.ac.uk/spm

## References

[B1] Amenedo E. Gutiérrez-Domínguez F. J. Mateos-Ruger S. M. Pazo-Álvarez P. (2014). Stimulus-locked and response-locked ERP correlates of spatial Inhibition of Return (IOR) in old age. *J. Psychophysiol.* 28 105–123. 10.1027/0269-8803/a000119

[B2] Bartolomeo P. Chokron S. Siéroff E. (1999). Facilitation instead of inhibition for repeated rightsided events in left neglect. *Neuroreport* 10 3353–3357. 10.1097/00001756-199911080-00018 10599844

[B3] Bourgeois A. Chica A. B. Valero-Cabré A. Bartolomeo P. (2013). Cortical control of inhibition of return: causal evidence for task-dependent modulations by dorsal and ventral parietal regions. *Cortex* 49 2229–2238. 10.1016/j.cortex.2012.10.017 23332817

[B4] Cheal M. Chastain G. (2002). Timing of facilitatory and inhibitory effects of visual attention. *Vis. Cogn.* 9 969–1002. 10.1080/13506280143000467 11294037

[B5] Chen Q. Fuentes L. J. Zhou X. (2010). Biasing the organism for novelty: a pervasive property of the attention system. *Hum. Brain Mapp.* 31 1146–1156. 10.1002/hbm.20924 20063302 PMC6871094

[B6] Doallo S. Lorenzo-Lopez L. Vizoso C. Holguín S. R. Amenedo E. Bara S. (2004). The time course of the effects of central and peripheral cues on visual processing: an event-related potentials study. *Clin. Neurophysiol.* 115 199–210. 10.1016/S1388-2457(03)00317-1 14706489

[B7] Dorris M. C. Klein R. M. Everling S. Munoz D. P. (2002). Contribution of the primate superior colliculus to inhibition of return. *J. Cogn. Neurosci.* 14 1256–1263. 10.1162/089892902760807249 12495530

[B8] Dukewich K. R. (2009). Reconceptualizing inhibition of return as habituation of the orienting response. *Psychon. Bull. Rev.* 16 238–251. 10.3758/PBR.16.2.238 19293089

[B9] Eimer M. (1994). “Sensory gating” as a mechanism for visuospatial orienting: electrophysiological evidence from trial-by-trial cuing experiments. *Percept. Psychophys.* 55 667–675. 10.3758/BF03211681 8058454

[B10] Fecteau J. H. Muñoz D. P. (2005). Correlates of capture of attention and inhibition of return across stages of visual processing. *J. Cogn. Neurosci.* 17 1714–1727. 10.1162/089892905774589235 16269108

[B11] Fuentes L. J. (2004). “Inhibitory processing in the attentional networks.” in *Cognitive Neuroscience of Attention* ed. Posner M. I. (New York, NY: Guilford Press), 45–55.

[B12] Gross J. Baillet S. Barnes G. R. Henson R. N. Hillebrand A. Jensen O. (2013). Good practice for conducting and reporting MEG research. *Neuroimage* 65 349–363. 10.1016/j.neuroimage.2012.10.001 23046981 PMC3925794

[B13] Gutiérrez-Domínguez F. J. Pazo-Álvarez P. Doallo S. Fuentes L. J. Lorenzo-López L. Amenedo E. (2014). Vertical asymmetries and inhibition of return: effects of spatial and non-spatial cueing on behavior and visual ERPs. *Int. J. Psychophysiol.* 91 121–131. 10.1016/j.ijpsycho.2013.12.004 24342058

[B14] Hilchey M. D. Dohmen D. Crowder N. A. Klein R. M. (2016). When is inhibition of return input-or output-based? It depends on how you look at it. *Can. J. Exp. Psychol.* 70 325–334. 10.1037/cep0000075 26654387

[B15] Hilchey M. D. Klein R. M. Satel J. (2014). Returning to “inhibition of return” by dissociating long-term oculomotor IOR from short-term sensory adaptation and other nonoculomotor “inhibitory” cueing effects. *J. Exp. Psychol.* 40 1603–1616. 10.1037/a0036859 24820438

[B16] Hopfinger J. B. Mangun G. R. (1998). Reflexive attention modulates processing of visual stimuli in human extrastriate cortex. *Psychol. Sci.* 9 441–447. 10.1111/1467-9280.00083 26321798 PMC4552358

[B17] Hopfinger J. B. Mangun G. R. (2001). Tracking the influence of reflexive attention on sensory and cognitive processing. *Cogn. Affect. Behav. Neurosci.* 1 56–65. 10.3758/CABN.1.1.56 12467103

[B18] Ille N. Berg P. Scherg M. (2002). Artifact correction of the ongoing EEG using spatial filters based on artifact and brain signal topographies. *J. Clin. Neurophysiol.* 19 113–124. 10.1097/00004691-200203000-00002 11997722

[B19] Ivanoff J. Klein R. M. (2001). The presence of a nonresponding effector increases inhibition of return. *Psychon. Bull. Rev.* 8 307–314. 10.3758/BF03196166 11495119

[B20] Ivanoff J. Klein R. M. (2006). Inhibition of return: sensitivity and criterion as a function of response time. *J. Exp. Psychol.* 32 908–919. 10.1037/0096-1523.32.4.908 16846287

[B21] Kalogeropoulou F. Woodruff P. W. Vivas A. B. (2015). Inhibition of return is not impaired but masked by increased facilitation in schizophrenia patients. *Neuropsychology* 29 10–16. 10.1037/neu0000092 24885452

[B22] Klein R. M. (2000). Inhibition of return. *Trends Cogn. Sci.* 4 138–147. 10.1016/S1364-6613(00)01452-2 10740278

[B23] Klein R. M. Hilchey M. D. (2011). “Oculomotor inhibition of return,” in *The Oxford Handbook of Eye Movements*, eds Liversedge S. P. Gilchrist I. Everling S. (Oxford: Oxford University Press), 471–492.

[B24] Lawrence L. M. Ciorciari J. Kyrios M. (2014). Cognitive processes associated with compulsive buying behaviours and related EEG coherence. *Psychiatry Res.* 221 97–103. 10.1016/j.pscychresns.2013.10.005 24239477

[B25] Lepsien J. Pollmann S. (2002). Covert reorienting and inhibition of return: an event-related fMRI study. *J. Cogn. Neurosci.* 14 127–144. 10.1162/089892902317236795 11970781

[B26] Lupiáñez J. (2010). “Inhibition of return,” in *Attention and Time*, eds Nobre A. C. Coull J. T. (Oxford: Oxford University Press), 17–34. 10.1093/acprof:oso/9780199563456.003.0002

[B27] Lupiañez J. Martín-Arévalo E. Chica A. B. (2013). Is inhibition of return due to attentional disengagement or to a detection cost? The detection cost theory of IOR. *Psicologica* 34 221–252.

[B28] Lupiáñez J. Milán E. G. Tornay F. J. Madrid E. Tudela P. (1997). Does IOR occur in discrimination tasks? Yes, it does, but later. *Percept. Psychophys.* 59 1241–1254. 10.3758/BF03214211 9401458

[B29] Maris E. (2012). Statistical testing in electrophysiological studies. *Psychophysiology* 49 549–565. 10.1111/j.1469-8986.2011.01320.x 22176204

[B30] Maris E. Oostenveld R. (2007). Nonparametric statistical testing of EEG-andMEG-data. *J. Neurosci. Methods* 164 177–190. 10.1016/j.jneumeth.2007.03.024 17517438

[B31] Michel C. M. Murray M. M. Lantz G. Gonzalez S. Spinelli L. de Peralta R. G. (2004). EEG source imaging. *Clin. Neurophysiol.* 115 2195–2222. 10.1016/j.clinph.2004.06.001 15351361

[B32] Moont R. Crispel Y. Lev R. Pud D. Yarnitsky D. (2012). Temporal changes in cortical activation during distraction from pain: a comparative LORETA study with conditioned pain modulation. *Brain Res.* 1435 105–117. 10.1016/j.brainres.2011.11.056 22192409

[B33] Nichols T. E. (2012). Multiple testing corrections, nonparametric methods, and random field theory. *Neuroimage* 62 811–815. 10.1016/j.neuroimage.2012.04.014 22521256

[B34] Pascual-Marqui R. D. (2002). Standardized low-resolution brain electromagnetic tomography (sLORETA): technical details. *Methods Find Exp. Clin. Pharmacol.* 24(Suppl D), 5–12. 12575463

[B35] Pastötter B. Hanslmayr S. Bäuml K. H. (2008). Inhibition of return arises from inhibition of response processes: an analysis of oscillatory beta activity. *J. Cogn. Neurosci.* 20 65–75. 10.1162/jocn.2008.20010 17919085

[B36] Posner M. I. (1980). Orienting of attention. *Q. J. Exp. Psychol.* 32 3–25. 10.1080/00335558008248231 7367577

[B37] Posner M. I. Cohen Y. (1984). “Components of visual orienting,” in *Attention and Performance X: Control of Language Processes*, eds Bouma H. Bouwhuis D. G. (Hove: Lawrence Erlbaum Associates Ltd), 531–556.

[B38] Posner M. I. Rafal R. D. Choate L. S. Vaughan J. (1985). Inhibition of return: neural basis and function. *Cogn. Neuropsychol.* 2 211–228. 10.1080/02643298508252866

[B39] Prime D. J. Ward L. M. (2004). Inhibition of return from stimulus to response. *Psychol. Sci.* 15 272–276. 10.1111/j.0956-7976.2004.00665.x 15043647

[B40] Prime D. J. Ward L. M. (2006). Cortical expressions of inhibition of return. *Brain Res.* 1072 161–174. 10.1016/j.brainres.2005.11.081 16445889

[B41] Rosen A. C. Rao S. M. Caffarra P. Scaglioni A. Bobholz J. A. Woodley S. J. (1999). Neural basis of endogenous and exogenous spatial orienting: a functional MRI study. *J. Cogn. Neurosci.* 11 135–152. 10.1162/089892999563283 10198130

[B42] Saha S. Nesterets Y. I. Tahtali M. Gureyev T. E. (2015). Evaluation of spatial resolution and noise sensitivity of sLORETA method for EEG source localization using low-density headsets. *Biomed. Phys. Eng. Express* 1:045206 10.1088/2057-1976/1/4/045206

[B43] Sapir A. Soroker N. Berger A. Henik A. (1999). Inhibition of return in spatial attention: direct evidence for collicular generation. *Nat. Neurosci.* 2 1053–1054. 10.1038/15977 10570480

[B44] Sohrabpour A. Lu Y. Kankirawatana P. Blount J. Kim H. He B. (2015). Effect of EEG electrode number on epileptic source localization in pediatric patients. *Clin. Neurophysiol.* 126 472–480. 10.1016/j.clinph.2014.05.038 25088733 PMC4289666

[B45] Song X. W. Dong Z. Y. Long X. Y. Li S. F. Zuo X. N. Zhu C. Z. (2011). REST: a toolkit for resting-state functional magnetic resonance imaging data processing. *PLoS One* 6:e25031. 10.1371/journal.pone.0025031 21949842 PMC3176805

[B46] Taylor T. L. Klein R. M. (2000). Visual and motor effects in inhibition of return. *J. Exp. Psychol.* 26 1639–1656. 10.1037/0096-1523.26.5.1639 11039490

[B47] Tian Y. Klein R. M. Satel J. Xu P. Yao D. (2011). Electrophysiological explorations of the cause and effect of inhibition of return in a cue–target paradigm. *Brain Topogr.* 24 164–182. 10.1007/s10548-011-0172-3 21365310

[B48] Tzourio-Mazoyer N. Landeau B. Papathanassiou D. Crivello F. Etard O. Delcroix N. (2002). Automated anatomical labeling of activations in SPM using a macroscopic anatomical parcellation of the MNI MRI single-subject brain. *Neuroimage* 15 273–289. 10.1006/nimg.2001.0978 11771995

[B49] van Koningsbruggen M. G. Gabay S. Sapir A. Henik A. Rafal R. D. (2010). Hemispheric asymmetry in the remapping and maintenance of visual saliency maps: a TMS study. *J. Cogn. Neurosci.* 22 1730–1738. 10.1162/jocn.2009.21356 19803692

[B50] Vivas A. B. Humphreys G. W. Fuentes L. J. (2003). Inhibitory processing following damage to the parietal lobe. *Neuropsychologia* 41 1531–1540. 10.1016/S0028-3932(03)00063-0 12849771

[B51] Vivas A. B. Humphreys G. W. Fuentes L. J. (2006). Abnormal inhibition of return: a review and new data on patients with parietal lobe damage. *Cogn. Neuropsychol.* 23 1049–1064. 10.1080/02643290600588400 21049367

[B52] Wascher E. Falkenstein M. Wild-Wall N. (2011). Age related strategic differences in processing irrelevant information. *Neurosci. Lett.* 487 66–69. 10.1016/j.neulet.2010.09.075 20933055

[B53] Wascher E. Tipper S. P. (2004). Revealing effects of noninformative spatial cues: an EEG study of inhibition of return. *Psychophysiology* 41 716–728. 10.1111/j.1469-8986.2004.00198.x 15318878

[B54] Wright R. D. Richard C. M. (2000). Location cue validity affects inhibition of return of visual processing. *Vis. Res.* 40 2351–2358. 10.1016/S0042-6989(00)00085-7 10927120

